# *Mycobacterium bovis* infections in slaughter pigs in Mubende district, Uganda: a public health concern

**DOI:** 10.1186/1746-6148-8-168

**Published:** 2012-09-21

**Authors:** Adrian Muwonge, Tone B Johansen, Edvardsen Vigdis, Jacques Godfroid, Francisco Olea-Popelka, Demelash Biffa, Eystein Skjerve, Berit Djønne

**Affiliations:** 1 Department of Food Safety and Infection Biology, Norwegian School of Veterinary Science, P.O. Box 8146, Dep., 0033, Oslo, Norway; 2 Norwegian Veterinary Institute, P.O. Box 750, N-0106, Oslo, Norway; 3 Department of Food Safety and Infection Biology, Section of Arctic Veterinary Medicine, Norwegian School of Veterinary Science, Stakkevollveien 23, 9010, Tromsø, Norway; 4 Department of Clinical Sciences College of Veterinary Medicine and Biomedical Sciences Colorado State University Fort Collins, Fort Collins, CO, 80523-1678, USA; 5 College of Medicine, University of Arizona, 1656 E. Mabel St, P.O. Box 245221, Tucson, AZ, 85724, USA

**Keywords:** Pigs, Spoligotype, MIRU-VNTR, M. bovis, Uganda

## Abstract

**Background:**

Bovine tuberculosis (TB) caused by *Mycobacterium bovis* is primarily a disease of ruminants, particularly cattle (*Bos primigenius)* and buffalo (*Syncerus caffer*), and is endemic in most developing countries. To date, studies done in Uganda have documented the prevalence of *M. bovis* in cattle, humans and wild life, in addition to non-tuberculous mycobacteria in pigs. Pigs are increasingly becoming an important component of the livestock sector and share the human ecosystem in rural Uganda. It is therefore of public health interest that they are not a source of human infections. As a follow up to previously published findings on mycobacteria in pigs, this study was aimed at investigating the occurrence and molecular characteristics of *M. bovis* detected in slaughter pigs in Mubende district, Uganda. One hundred fifty mesenteric lymph nodes with lesions suggestive of mycobacterial infections were collected from approximately one thousand slaughtered pigs in Mubende district over a period of five months. The isolation and identification of *M. bovis* was done using conventional mycobacteriological methods. Mycobacteria belonging to the *Mycobacterium tuberculosis* complex (MTC) were identified to species level using deletion analysis. Molecular typing was done using Spoligotyping and MIRU-VNTR analysis. Molecular data were analysed and interpreted using MIRU-VNTR *plus*, SpolDB4.0 and the *Mycobacterium bovis* spoligo database.

**Results:**

Of the examined animals, one boar and two sows from Madudu Sub County were infected with *M. bovis* which presented as lesions of a deep yellow colour and a grit-like texture in the mesenteric lymph nodes. This represents 2% (3/150) of the lymph nodes where lesions suggestive of mycobacterial infections were detected. Molecular analysis revealed that the isolates from the infected pigs showed identical MIRU-VNTR profile and spoligotype (SB1469).

**Conclusions:**

This is the first study documenting the occurrence of *M. bovis* in slaughter pigs in Uganda, revealing that one in fifty slaughter pigs with suspected lesions in mesenteric lymph nodes were infected*.* Molecular analysis revealed that the isolates were identical, showing a spoligotype previously reported from humans and cattle in the north eastern part of the Uganda cattle corridor. This finding is of public health importance, therefore there is a need for close cooperation between medical and veterinary professionals in designing and implementing control and prevention measures that safeguard the public from this potential source of zoonotic TB in Uganda.

## Background

*Mycobacterium bovis* (*M. bovis*) is the etiological agent of bovine tuberculosis (TB). Bovine TB is a recrudescent zoonosis whose eradication has eluded some of the least and most developed countries in the world. In most developing countries, bovine TB is endemic and there is little information available regarding the relationship between *M. bovis* infection in livestock, wildlife and the disease in humans [1,2]. The World Health Organization recently classified human TB caused by *M. bovis* as a “neglected zoonosis” [2]. Moreover, areas where bovine TB is endemic overlap with countries afflicted by high HIV/AIDS and tuberculosis co-infection rates*.* The added exposure to bovine TB presents a significant risk for developing *M. bovis* infection among humans [3]. There are some studies providing insight into the significance of *M. bovis* infection in humans in developing countries, however, the true extent of this problem remains largely unknown [2,3].

Humans can be infected by *M. bovis* orally, via inhalation of aerosolized particles containing *M. bovis* from infected animals [3] or through person to person transmission [4]. However, the most common route of infection with *M. bovis* is through the oral route by consumption of contaminated dairy products or undercooked animal products (i.e. from bovines and goats) [3,5,6]. *M. bovis* has the ability to survive for long periods in soil and slurry [7], a phenomenon which has recently been reaffirmed by a quantification assay that detected *M. bovis* in environmental samples [8]. This can represent yet another source of *M. bovis* for humans and animals, especially in bovine TB endemic areas.

Porcine TB caused by *M. bovis* has been reported in Argentina, South Africa and West Africa [1,9-11]. In South Africa, *M. bovis* accounted for 2.5% of isolated mycobacteria from pigs with pathological lesions [10]. Pigs can become infected from cattle by the oral route [1], and outbreaks have been associated with infected yards or buildings contaminated with infected faecal material, feeding piglets with infected cattle milk and contact with wild life [9-12]. Unlike in the past where pigs were considered an epidemiological dead end in transmission of bovine TB, reports in Spain and Portugal show a multi-directional cross contamination between domestic pigs and wild boars [13,14].

Pigs are increasingly becoming an important component of the livestock sector and human ecosystems in Uganda [15], therefore it is of public health interest to know if they are infected by zoonotic agents such as *M. bovis*[9,10]. Infected pigs are reported to present with progressive lesions of defined tubercles in the lungs, spleen and lymph nodes in the thoracic and/or abdominal cavity usually visible at inspection [10]. Routine abattoir meat inspections is the only economically feasible food safety tool that can be used in resource limited settings [10], however routine inspection is reported to have a low sensitivity for detecting tuberculosis lesions [16,17]. A recent study on slaughter pigs in Uganda also revealed that up to 31% of slaughtered pigs without visible lesions were harbouring non-tuberculous mycobacteria [18]. The sensitivity of routine inspection is reported to be affected by the frequency, dispersion and size of the lesions in addition to abattoir-environment factors like lighting and speed of flow system [19]. A combination of human factors like knowledge, experience, motivation, diligence, autonomy, and workload of the individuals conducting the examination can also significantly affect the outcomes of a routine meat inspection [19].

Molecular epidemiological tools have been used to characterise isolates of *M. bovis*, in order to decipher the dynamics or distribution and spread of bovine TB in different hosts. In that regard, spoligotyping and MIRU-VNTR are highly recommended tools for molecular epidemiological investigations and transmission chain tracing [1,20,21]. In Uganda, close contact exists between humans, pigs, cattle, wildlife and the environment. Earlier studies have characterised isolates of *M. bovis* from cattle and humans in the cattle corridor, revealing identical spoligotypes [22,23]. There are reports documenting the prevalence of non-tuberculous mycobacteria from slaughtered pigs in the Mubende district [18,24], but the role of pigs in the epidemiology of *M. bovis* had not been explored. This study was thus aimed at contributing to the knowledge of the epidemiology of *M. bovis* infections in Uganda, by investigating the occurrence and molecular characteristics of *M. bovis* from slaughter pigs, and by comparing these isolates with *M. bovis* detected from humans and cattle in earlier studies.

## Material and method

### Study site

**Figure 1:**
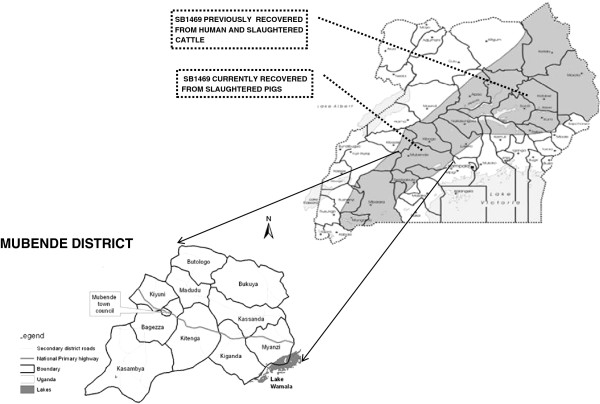
**Map of the Uganda cattle corridor showing Mubende district and sub counties, illustrating where *Mycobacterium bovis *spoligotype SB1469 has previously been recovered from humans and slaughtered cattle [**[23]**] and slaughtered pigs (present study).**

Mubende is located in the central region (000 33' 27''N, 310 23' 42''E) of Uganda. It is divided into two counties namely; Buwekula and Kassanda which are further divided into 10 Sub Ccounties; Bagezza (urbanized sub county), Butologo, Kasambya, Kitenga, Kiyuni, Madudu, Bukuya, Kassanda, Kiganda and Myanzi (Figure 1). Mubende is inhabited by approximately 750,000 people, 60% of whom live below the poverty line in population dense urban and peri-urban areas, where the prevalence of HIV is reported to be as high as 18% [25,26]. The district lies in the typical pastoral Uganda cattle corridor, and is endowed with livestock like cattle (35,000), goats (12,000), sheep (6,000), pigs (80,000) and chicken (360,000) [25,26]. Madudu and Kiyuni sub counties are home to the largest proportion of the pig population in the district majority of which are reared on free range [26,27]. Mubende district is also blessed with wild life especially wild swine at the south eastern corner of the district in proximity with Lake Wamala [28].

### Study animals

The present study was carried out between February and June 2011 as a follow up to a previous study conducted by Muwonge et al. [24]. The earlier study documented the prevalence of non- tuberculous mycobacteria in slaughtered pigs based on the detection of acid fast bacteria and molecular identification of mycobacteria from cervical lymph nodes in the same area [18,24].

In the present study, an approximate total of 1000 pig carcasses slaughtered at the age of one year or older were inspected at thirty one slaughter houses which were randomly selected, as described in [21]. They slaughtered about 200 animals each month [29], and were randomly visited for a convenient sampling of mesenteric lymph nodes with gross pathological lesions during a period of five months. The average number of pigs slaughtered varied at slaughter house level, with the majority of slaughter houses in Madudu, Kiyuni, Bagezza and Mubende town council slaughtering 5–8 pigs while the rest slaughtered 3–5 pigs per day.

### Sampling and isolation of Mycobacteria

Mesenteric lymph nodes that showed the following gross pathological picture were collected; increased size, colour changes, high vascularisation, granulation and/or caseous texture. One hundred and fifty mesenteric lymph nodes were collected and shipped to the BSL3 laboratory at the Norwegian Veterinary institute in Oslo for analysis. Data on Sub County, rearing method and sex of the animal were recorded for each sample (Table 1).

**Table 1:** **The distribution of *Mycobacterium bovis *recovered from lymph nodes with lesions in slaughtered pigs in Mubende district**

**Variable**	**Label**	**Number sampled**	**No with *M. bovis***	**Percentage %**
Pigs	Sampled lymph nodes	150	3	2
Sex	Male	91	1	1.09
	Female	59	2	3.4
Rearing Method	Free range	82	3	3.7
	Teathered	32	0	-
	Housed	36	0	-
Source sub county	Madudu	44	3	6.8
	Mubende town council	16	0	-
	Bagezza	25	0	-
	Bukuya	11	0	-
	Kiyuni	16	0	-
	Butologo	11	0	-
	Kiganda	7	0	-
	Myanzi	6	0	-
	Kitenga	4	0	-
	Kassanda	4	0	-
	Kasambya	6	0	-

Approximately 3 grams of the lymph nodes was ground with sterile sand and decontaminated with oxalic acid as described in Muwonge et al. [24]. Finally, 2–3 drops were transferred to both Stonebrink with pyruvate and Middelbrook media and incubated for 8 weeks at 37°C. The media were monitored weekly for growth. Acid fast bacteria, as demonstrated by Ziehl-Neelsen staining, were identified as *Mycobacterium* spp, and further identified.

### Mycobacterial DNA extraction and molecular analysis

Genomic DNA was extracted by heat inactivation of culture material dissolved in TE buffer and the supernatant was used directly as template. Sequencing of the 16S rDNA gene was performed as described by Muwonge et al. [18].

Identification of isolates belonging to the *M. tuberculosis* complex (MTC) was performed by genomic region of difference analysis as previously described [29] at the Norwegian Veterinary Institute. A set of primers including RD1, RD4, RD9 and RD12 were used [30]. Spoligotyping and MIRU-VNTR were performed by Genoscreen^©^. For spoligotyping; amplification of the spacers was done using the primers DRa and DRb, which enable amplification of the whole DR region [31]. For MIRU-VNTR, the standardized protocol using 15 loci as described by Supply et al. 2006 were used [32]. The results were reported in Roman numerals representing the number of repeats per loci. *M. bovis* BCG and *M. tuberculosis* strain H37Rv were used as positive controls and water as a negative control for both methods.

### Data analysis

Molecular results from Genoscreen^©^ in France and the Norwegian Veterinary Institute were entered and validated in Excel® 2007. These were then copied into MIRU-VNTR *plus* to compare with strains in the data base. The same data set was compared against the SpolDB4.0 and *M. bovis* spoligo data bases available at http://www.pasteur-guadeloupe.fr:8081/SITVITDemo/ and http://www.mbovis.org/ respectively.

### Ethical considerations

Scientific and ethical clearance for this study was obtained from the Uganda National Council for Science and Technology (UNCST). The research ethics committee found this study to be scientifically and ethically in accordance with the requirements and therefore was approved with reference number HS 879.

## Results

### Culture results

Isolates were verified as belonging to the *M. tuberculosis* complex based on typical growth on culture media, positive result on ZN staining and by 16S rDNA sequencing. Deletion analysis confirmed the isolates as *M. bovis*, based on presence of RD1 and absence of RD4, RD9 and RD12 [30].

*M. bovis* was detected from three (2%) of the 150 examined lymph nodes with lesions compatible with tuberculosis. The lymph nodes originated from three different pigs. The isolates were recovered from one boar and two sows in Madudu Sub County (Table 1). It is noteworthy that some rapid growing non-tuberculous mycobacteria where recovered from the sampled lymph (unpublished results).

### Gross appearance of lymph nodes

**Figure 2:**
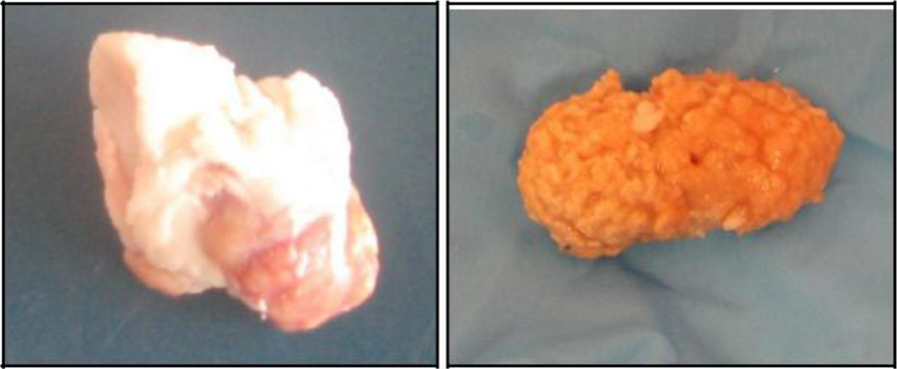
**Gross pathological appearance of mesenteric lymph nodes from which *Mycobacterium bovis *was isolated.**

The results show that two of the mesenteric lymph nodes, each recovered from a different pig, from which *M. bovis* was recovered, showed typical lesions of tuberculosis. These were characterized by caseocalcerous tubercle appearance with grit-like particles embedded within deep yellow remnants of the lymph node (Figure 2). The last lymph node appeared abnormal but not as distinctive.

### Molecular characteristics of *M. bovis* isolates

All the three isolates were identical on both MIRU-VNTR analysis and spoligotyping (Table 2). Based on the latter, the isolates lacked hybridization at position 3–7, 9, 16, 18–22 and 39–43. This spoligo pattern was registered in the *M. bovis* spoligo database as SB1469.

**Table 2:** **MIRU-VNTR and Spoligotype of *Mycobacterium bovis *isolated from mesenteric lymph nodes of slaughtered pigs in Mubende district, Uganda**

**ID**	**Source**	**Sex**	**Host**	**MIRU-VNTR (15 standard loci)**	**Spoligotype**
66c	Madudu	M	Pig	263223332453231	
67	Madudu	F	Pig	263223332453231	
71c1	Madudu	F	Pig	263223332453231	

F = female, M = male.

## Discussion

This study documents the occurrence of *M. bovis* in slaughtered pigs in Uganda for the first time. The findings are of public health concern in this predominantly pig rearing and pork consuming population with a known high HIV prevalence.

The results revealed that 2% of the mesenteric lymph nodes with lesions submitted for analysis were infected with *M. bovis*, which is approximately similar to the 2.5% prevalence detected in South Africa [10]. Typical macroscopic TB pathology, characterized by a deep yellow colouring of the lymph node with a grit-like texture on culture processing, was visible in two of the mesenteric lymph nodes. This pathological picture is in concordance with that reported by Cousin et al. [10]. The actual prevalence of *M. bovis* infections in pigs might be higher than detected in this study given that a convenient sampling was used which might have missed infected lymph nodes that had not yet developed lesions. Additionally, growth of non-tuberculous mycobacteria might have concealed the presence of *M. bovis* in some samples.

Epidemiologically, pigs are considered spill over hosts in *M. bovis* transmission [13,14] therefore in conventional epidemiological studies they would not be the focal host of investigation. Some studies have however shown that depending on the rearing system in use, pigs can be used as sentinels for other hosts [12]. Therefore, as there is no study published on the prevalence of *M. bovis* in domestic and wild animals in Mubende district, this could be an indicator of a higher prevalence among cattle and/or wild boars in the study area. The public health conundrum is that although routine abattoir meat inspection is the economically feasible food safety tool in resource limited settings, it is reported to have a low sensitivity for detecting tuberculosis lesions [16-18]. In Mubende district, this situation is likely to be compounded by unreliable routine meat inspection and veterinary extension services [27,29]. This could imply that a considerable proportion of the human population can be exposed to mycobacteria of porcine origin if and when undercooked pork is consumed.

Reports in Africa have indicated that pigs are infected with *M. bovis* through direct contact with infected cattle or by feeding piglets with infected milk from cattle [5,9]. In southern Europe, domestic pigs are reported to be infected by wildlife reservoir hosts like the wild boar [13,14]. In Mubende, both sources of infection are plausible, given that the district lies in the Uganda cattle corridor where *M. bovis* prevalence has been documented [22,23] and the presence of bush pigs and warthogs at the south western corner of the district [28]. All the *M. bovis* isolates were recovered from Madudu Sub County. This apparent geographic predilection could be due to the high pig population in Madudu which inherently formed the biggest proportion of the sample.

Molecular analysis revealed that all the three isolates were identical on spoligotyping and MIRU-VNTR analysis and although we could not establish if these pigs were from the same farm, their identical genotype suggests a common source of infection. The spoligo patterns were identified as SB1469, previously reported from cattle and humans in Karamoja in the north eastern part of the Uganda cattle corridor [22,23]. Both Karamoja and Mubende lay in the Uganda cattle corridor, which is characterized by a high cattle population and high mobility of people and livestock. This seem to indicate that the presently described spoligotype could be widely prevalent in the Uganda cattle corridor, although SB1469 was not among the spoligotypes reported in Kampala [33] where most of the slaughtered cattle originate from the south-western part of the Uganda cattle corridor.

## Conclusions

This is the first study documenting the occurrence of *M. bovis* in slaughter pigs in Uganda, revealing that one in fifty slaughter pigs with suspected lesions in mesenteric lymph nodes were infected*.* Molecular analysis revealed that the isolates were identical, showing a spoligotype previously reported from humans and cattle in the north eastern part of the Uganda cattle corridor. This finding is of public health importance, therefore there is a need for close cooperation between medical and veterinary professionals in designing and implementing control and prevention measures that safeguard the public from this potential source of zoonotic TB in Uganda.

## Competing interests

This was purely research work and therefore to the best of our knowledge there was no competing interest.

## Authors’ contributions

AM: contributed to the conception, design, and data collection, laboratory work, drafting and writing of the manuscript. TBJ: contributed to laboratory work, data analysis and drafting of the manuscript. VE, DB, FOP: contributed to the laboratory analysis and drafting of the manuscript. JG, ES and BD: contributed to the acquisition of funds, design of study and drafting of the manuscript. All authors have read and approved the final manuscript.
